# Multi‐omic analysis identifies biological processes underlying progressive interstitial lung disease in systemic sclerosis

**DOI:** 10.1111/febs.70177

**Published:** 2025-07-07

**Authors:** Selena Bouffette, Perrine Soret, Alice Cole, Emmanuel Nony, Pierre Barbier Saint Hilaire, Grégory Leclerc, Lamine Alaoui, Audrey Aussy, Isabelle Wehrle, Aude Le Gall, Mark Coulon, Marc Pallardy, Voon Ong, Jeanne Allinne, Philippe Moingeon, David Abraham, Christopher P. Denton

**Affiliations:** ^1^ Servier R&D Institute Gif‐sur‐Yvette France; ^2^ Inflammation Microbiome and Immunosurveillance Université Paris‐Saclay, Inserm Orsay France; ^3^ Division of Medicine, Centre for Rheumatology University College London (UCL) UK

**Keywords:** interstitial lung fibrosis, lung fibrosis, multi‐omics, scleroderma, systemic sclerosis

## Abstract

Systemic sclerosis (SSc) is a rare autoimmune connective tissue disorder, and its primary cause of mortality is interstitial lung disease (ILD). This study aimed to identify markers in patients with SSc that are associated with ILD progression. In total, 52 SSc patients and five healthy volunteers (HVs) were included. Patient plasma samples were available for measurement of soluble mediators by metabolomics, proteomics, and cytokine quantification. Gene expression profiling was performed on patients' whole blood and skin biopsies, and immunophenotyping was carried out on peripheral blood mononuclear cells. Comparisons were made between patients with progressive ILD, those with no ILD, and HVs. Our results confirm the involvement of pro‐inflammatory mechanisms in SSc‐related ILD, with elevated type 1 interferon (IFN1), fractalkine (CX3CL1), and C‐C motif chemokine 2 (CCL2), as well as the profibrotic markers C‐X‐C motif chemokine 17 (CXCL17), thrombospondin (THBS), and latent transforming growth factor beta‐binding protein 1 (LTBP1). At the cellular level, lower inflammatory activity was observed in SSc‐ILD patients, which may be due to ongoing immunosuppressive therapies. ILD progression is associated with a significant increase in plasma levels of cytoskeletal proteins and lipids, notably triglycerides. To our knowledge, this is the first study using an innovative approach to compare SSc patients with ILD to those without ILD. Our study was performed on well‐characterized patients, from which we gathered insightful comparative data offering a multi‐level biological picture of SSc‐related ILD. A novel finding of our study is the correlation between elevated triglyceride levels and ILD progression, possibly linked to fibrogenesis through the role of triglycerides in endoplasmic reticulum stress.

## Graphical abstract

Systemic sclerosis (SSc) is a rare autoimmune disease, and lung complications (ILD) are the main cause of death. This study compared SSc patients with and without lung disease to healthy volunteers. We found increased inflammation, specific proteins, and higher triglyceride levels linked to lung disease progression. These findings suggest triglycerides may play a role in lung damage, offering new insights into how SSc‐related lung disease develops.

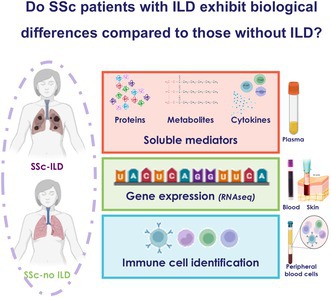

**Abbreviations**
ALB: albumin
APOB: apolipoprotein B
APOC3: apolipoprotein C3
APOH: apolipoprotein H
C9: complement component 9
CCL2: C‐C motif chemokine 2
CX3CL1: fractalkine
CXCL17: C‐X‐C motif chemokine 17
DLco: diffusing capacity for carbon monoxide
ECM: Extracellular Matrix
FGA: fibrinogen alpha chain
FVC: Forced Vital Capacity
Gal‐3: Galectin‐3
Gca: glycocholic acid
Gcdca: glycochenodeoxycholic acid
GSEA: Gene Set Enrichment Analysis
HLH: hemophagocytic lymphohistiocytosis
HP: haptoglobin
HRCT: High Resolution Computed Tomography
HV: Healthy Volunteer
IFN1: type 1 interferon
ILD: Interstitial Lung Disease
IPA: Ingenuity Pathway Analysis
IPF: Idiopathic Pulmonary Fibrosis
LPA: lysophosphatidic acid
LTBP1: latent transforming growth factor beta‐binding protein 1
ORM: orosomucoid protein
PBMC: peripheral blood mononuclear cell
PDGF: Platelet‐derived growth factor
SSc: Systemic Sclerosis
TGF‐β1: Transforming Growth Factor beta 1
THBS: thrombospondin
tSNE: t‐distributed stochastic neighbor embedding

## Introduction

Systemic sclerosis (SSc) is a rare autoimmune connective tissue disorder, characterized by tissue fibrosis, which impairs the ability of affected organs (skin, gastrointestinal tract, kidneys, heart and lungs) to perform their essential functions [1, 2]. It is plausible that SSc exemplifies an aberrant tissue repair process with a dysregulated immune response and an excessive extracellular matrix deposition by myofibroblasts, resulting in tissue scarring and fibrosis. This process occurs in the context of tissue injury that may originate in the endothelial or epithelial compartment [3, 4, 5].

Interstitial lung disease (ILD), a form of lung fibrosis, is the major complication of SSc. It has a prevalence of up to 80% among SSc patients, and it is the main cause of mortality (25–30%) [2, 6]. ILD severity ranges from mild to severe and can progress or not through the course of the disease [7, 8]. SSc‐ILD is a heterogeneous condition, more common among patients with anti‐topoisomerase autoantibodies, diffuse skin involvement, and of male gender [8]. Treatment strategies for SSc include immunosuppressive therapies combined with supportive care to manage symptoms. A first therapy to be approved for ILD treatment is Nintedanib, a tyrosine kinase inhibitor (FDA approval in 2020) [9], which has shown some efficacy in reducing forced vital capacity (FVC) decline. However, there is still no disease‐modifying treatment to improve ILD [10].

Previous studies have compared SSc patients to healthy volunteers with the aim to discern biological changes underlying fibrosis. They have mainly been performed on blood and skin samples that are generally more available than bronchoalveolar lavage fluid or lung tissue biopsies. Few of these studies have specifically compared SSc patients with ILD to those without. Such an approach may identify novel risk markers for the development or progression of ILD. Currently, there is an unmet need to identify patients at risk for progression of SSc‐ILD to optimize treatment initiation and extend current indicators of progression that focus on decline in FVC or radiologic findings [11].

In this context, we carried out an exploratory study, assessing well clinically characterized SSc patients with ILD from retrospective, prospective, and cross‐sectional cohorts, using integrated multi‐omic methods to evaluate biological changes in blood and skin samples. We aimed to identify markers that are associated with ILD progression in established SSc disease.

## Results

Our results highlight discrete biological differences within SSc and identify features associated with the presence of clinically significant progressive ILD. A synthesis of our results is shown in Table S1.

### Study participants

Fifty‐two SSc patients were divided into three cohorts:

- Cohort 1 (prospective): 9 SSc‐ILD and 3 SSc‐no ILD patients with longitudinal follow‐up over 6 months and 2 collection timepoints.
- Cohort 2 (retrospective): 20 SSc‐ILD and 10 SSc‐no ILD patients with longitudinal follow‐up over an average of 5 years and 3 collection timepoints.
- Cohort 3 (cross‐sectional): 10 patients with established SSc and presence (*n* = 5) or absence (*n* = 5) of ILD, at a single collection timepoint.

5 HVs were also included in our study.

The main patient characteristics are shown in Table 1.

**Table 1:** Summary of Systemic Sclerosis patient characteristics and available clinical data. Ab, antibody; ILD, interstitial lung disease; SSc, systemic sclerosis.

			Cohort n°1 (prospective)		Cohort n°2 (retrospective)		Cohort n°3 (cross‐sectional)	
			SSc‐ILD (*n* = 9)	SSc‐noILD (*n* = 3)	SSc‐ILD (*n* = 20)	SSc‐noILD (*n* = 10)	SSc‐ILD (*n* = 5)	SSc‐noILD (*n* = 5)
Demography								
Age		Median [Q1;Q3]	57 [46–65]	69 [67–74]	70 [59–76]	67 [64–69]	55 [51–77]	71 [67–76]
Gender	Female	*n* (%)	8 (89%)	1 (33%)	14 (70%)	7 (70%)	3 (60%)	4 (80%)
Subtype	Diffuse	*n* (%)	8 (89%)	2 (89%)	14 (70%)	2 (20%)	3 (60%)	1 (20%)
	Limited	*n* (%)	1 (11%)	1 (33%)	6 (30%)	8 (80%)	2 (40%)	4 (80%)
Disease duration (years)		Median [Q1;Q3]	3.0 [2.0–9.0]	2.0 [1.0–2.5]	–	–	14 [12–14]	15 [15–16]
Comorbidities								
Hypothyroidism		*n* (%)	1 (13%)		2 (10%)	2 (20%)	–	–
Osteoporosis		*n* (%)	1 (13%)	–	3 (15%)	–	–	–
Ischaemic heart disease		*n* (%)	1 (13%)	1 (33%)	2 (10%)	2 (20%)	–	–
Primary sclerosing cholangitis		*n* (%)	–	–	1 (5%)	1 (10%)	–	–
Chronic Kidney disease		*n* (%)	1 (13%)	–	3 (15%)	–	–	–
Atrial fibrillation		*n* (%)	1 (13%)	–	2 (10%)	–	–	–
Hypertension		*n* (%)	2 (22%)	1 (33%)	3 (15%)	1 (10%)	–	–
Asthma		*n* (%)	2 (22%)	–	–	–	–	–
Medication								
Mycophenolate		*n* (%)	7 (78%)	2 (67%)	14 (70%)	1 (10%)	3 (60%)	–
Methotrexate		*n* (%)	–	–	3 (15%)	–	1 (20%)	–
Cyclophosphamide		*n* (%)	1 (11%)	–	3 (15%)	–	–	–
Rituximab		*n* (%)	2 (22%)	–	1 (5%)	–	–	–
Hydroxychloroquine		*n* (%)	2 (22%)	–	–	–	2 (40%)	1 (20%)
Nintedanib		*n* (%)	1 (11%)	–	–	–	–	–
Tocilizumab		*n* (%)	2 (22%)	1 (33%)	–	–	–	–
Prednisolone		*n* (%)	–	1 (33%)	–	–	1 (20%)	–
Antibodies positivity profiles								
Anti‐centromere Ab		*n* (%)	7 (78%)	3 (100%)	6 (30%)	3 (30%)	–	4 (80%)
Anti‐topoisomerase Ab		*n* (%)	7 (78%)	1 (33%)	11 (55%)	–	4 (80%)	–
RNA‐polymerase III Ab		*n* (%)	1 (11%)	1 (33%)	3 (15%)	2 (20%)	–	1 (20%)
Anti‐PmScl antibodies		*n* (%)	–	–	–	–	1 (20%)	–

### Increased interferon (IFN) gene signature in blood and skin is associated with SSc‐ILD

**Fig. 1:**
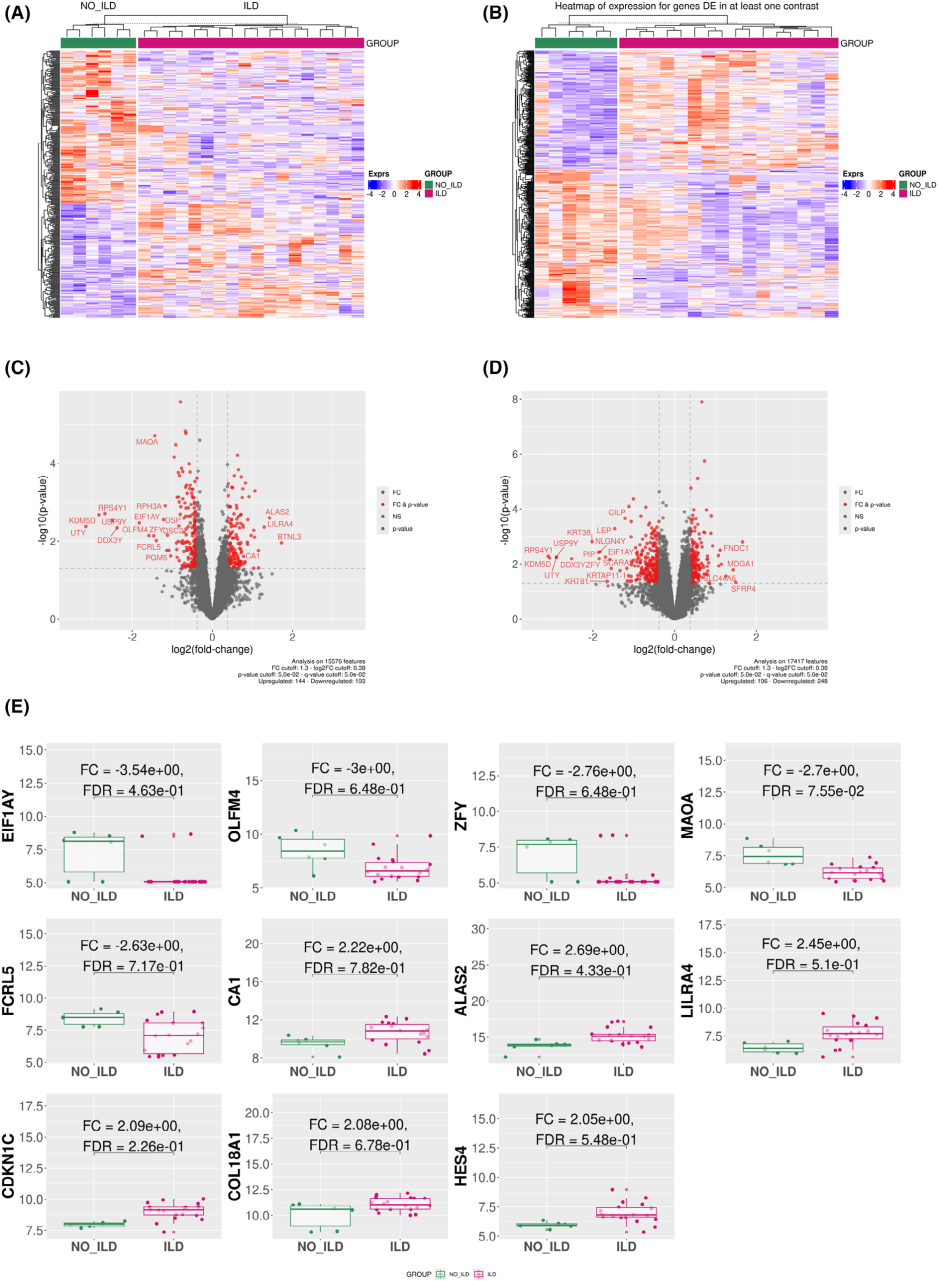
Comparative gene expression profiling of whole blood and skin biopsies from SSc‐ILD patients (*n* = 9) and SSc‐no ILD patients (*n* = 3). (A, B) Heatmaps of transcriptomic analysis of whole blood and skin (respectively) showing an upregulated IFN gene signature. (C, D) Volcano plots of differentially expressed genes from whole blood and skin (respectively) transcriptomic data. (E) Boxplots of the 11 most differentially expressed genes in the blood of SSc‐ILD patients vs. SSc‐no ILD patients. Results in E are depicted as boxes and whiskers plots defined by minimal, maximal, and median values in each dataset. The upper whisker extends from the hinge to the largest value no further than 1.5 * IQR from the hinge (where IQR is the interquartile range or distance between the first and third quartiles). The lower whisker extends from the hinge to the smallest value at most 1.5 * IQR of the hinge. Data beyond the end of the whiskers are called outlying points and are plotted individually. Statistical analyses were performed using limma analysis. ILD, interstitial lung disease; IFN, interferon; IQR, interquartile range; SSc, systemic sclerosis.

**Fig. 2:**
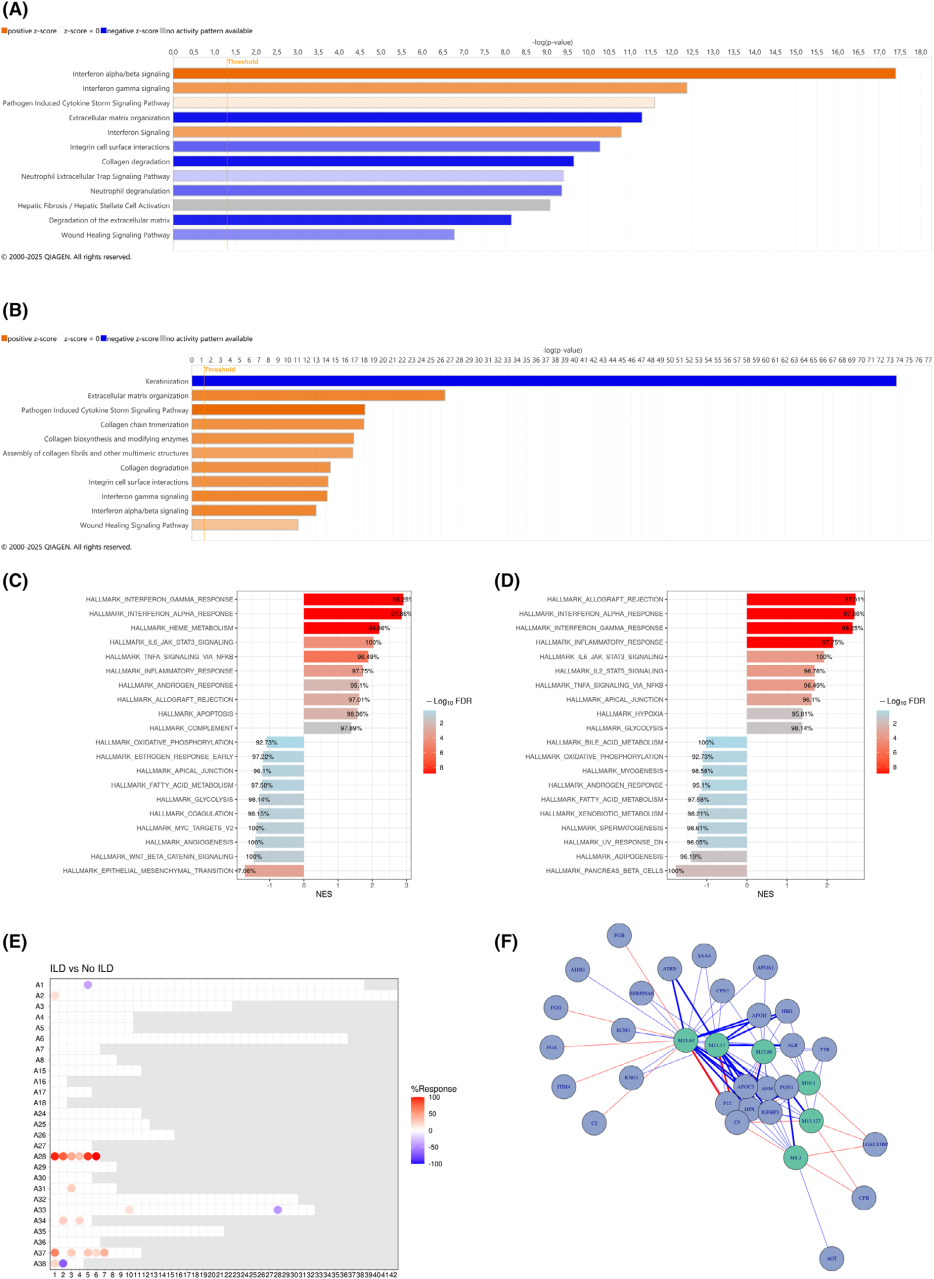
Comparative gene expression profiling of whole blood and skin biopsies from SSc‐ILD patients (*n* = 9) and SSc‐no ILD patients (*n* = 3). (A, B) Pathway analyses performed on transcriptomic data from the patient's whole blood and skin (respectively) using IPA. (C, D) GSEA pathway analysis of whole blood and skin transcriptomic data (respectively) in SSc‐ILD vs SSc‐no ILD patients. (E) Enrichment analysis performed on transcriptomic data resulting in the enrichment of 6 interferon modules (Altman *et al*., 2021) [4]. (F) Statistical correlation carried out between the 6 enriched interferon modules and the proteomic data. A positive correlation (red line) was identified between interferon modules and C9 and FGA, and a negative correlation (blue line) with APOH, APOC3, and ALB. ALB, albumin; APOC3, apolipoprotein C3; APOH, apolipoprotein H; C9, complement component 9; FGA, fibrinogen alpha chain; GSEA, gene set enrichment analysis; IFN, interferon; ILD, interstitial lung disease; IPA, Ingenuity Pathway Analysis; IQR, interquartile range; SSc, systemic sclerosis.

From the transcriptomic analysis of whole blood and skin biopsies of 12 SSc patients (Cohort 1), 57 773 transcripts were detected in the whole blood and the skin of patients, of which 15 600 transcripts were used for analysis in whole blood and 17 441 transcripts in the skin. A significant increase in the IFN gene signature was observed in the blood and skin of SSc‐ILD patients compared to SSc‐no ILD patients (Fig. 1A–E). Two separate pathway analyses of the datasets were performed, using IPA and GSEA (using the IPA and HALLMARK databases, respectively). An upregulation of inflammatory pathways (IFN‐α and ‐γ, heme, IL6/Jak/Stat3 and cytokine storm signaling) was observed in SSc‐ILD blood and skin compared to SSc‐no ILD blood (Fig. 2A–F). In SSc‐ILD blood, a downregulation of extracellular matrix‐related pathways (epithelial‐mesenchymal transition, ECM organization and collagen degradation) was observed (Fig. 2A), while SSc‐ILD skin showed very significant downregulation of the keratinization pathway, alongside an upregulation of collagen and extracellular matrix‐related pathways (Fig. 2B). A significant enrichment was observed for SSc‐ILD blood transcripts in the six interferon module sets reported by Altman *et al*. (2021) (Fig. 2E). The enriched IFN modules positively correlate to increased C9 and FGA protein levels, which are associated with inflammation. They also negatively correlate to reduced levels of APOH (altering platelet agglutination), APOC3 (delaying the catabolism of triglyceride‐rich particles), and ALB (albumin, main protein of human blood plasma, binds fatty acids) (Fig. 2F).

### Dysregulated protein and lipid metabolism relevant to vasculopathy and inflammation is associated with lung fibrosis in SSc

**Fig. 3:**
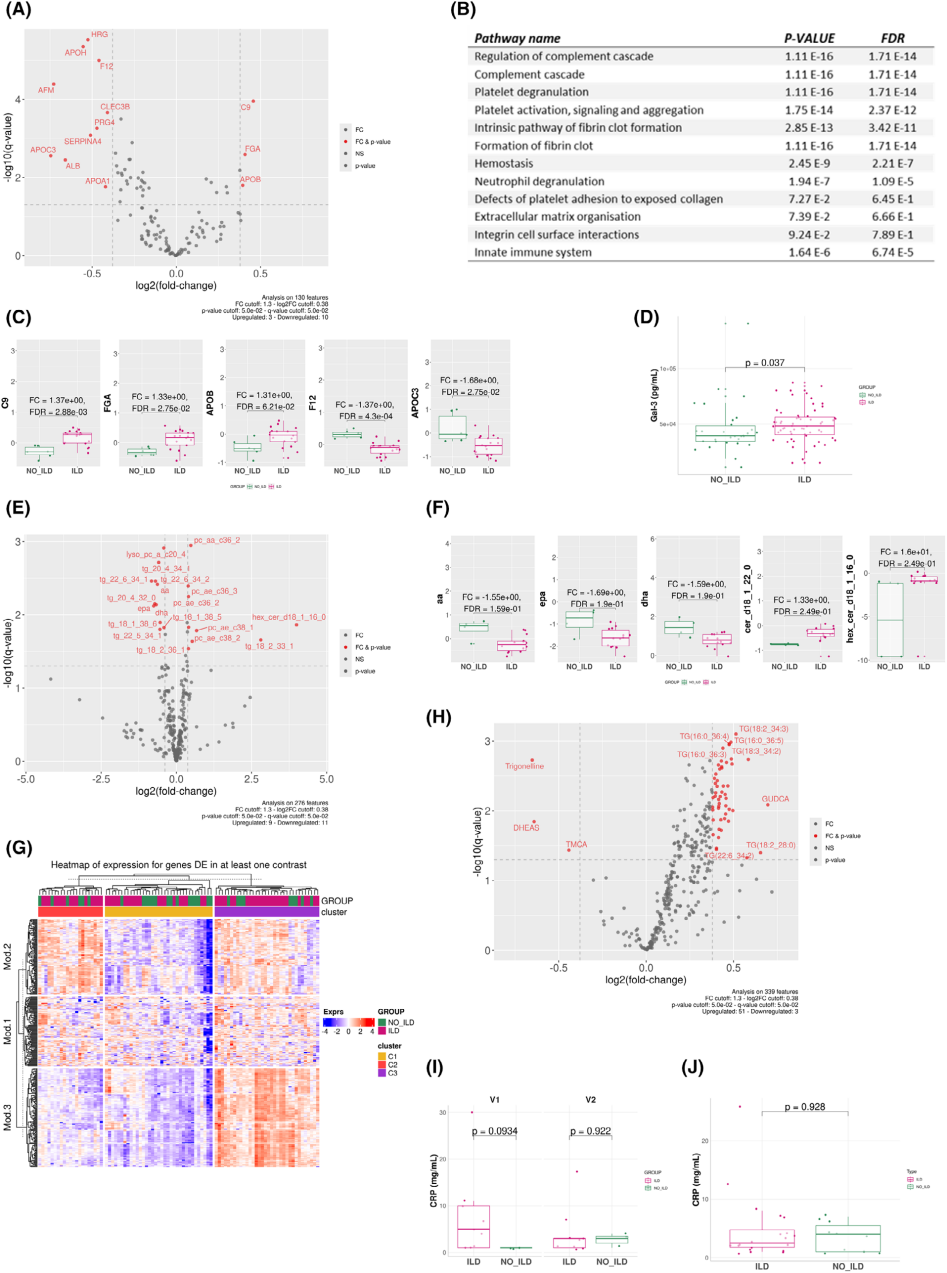
Profiling of plasma proteins and metabolites of SSc‐ILD patients compared to SSc‐no ILD patients. (A) Volcano plot of differentially expressed proteins in the plasma of 9 SSc‐ILD patients vs 3 SSc‐no ILD patients. (B) Reactome pathway analysis results of upregulated proteins. (C) Examples of 5 proteins whose levels are most significantly increased in SSc‐ILD patients' plasma. (D) Results of an ELLA assay of Galectin‐3, showing increased circulating levels in the plasma of SSc patients. (E) Volcano plot of differentially expressed metabolites in the plasma of 9 SSc‐ILD patients vs 3 SSc‐no ILD patients. (F) Examples of 5 metabolites whose levels are significantly different between SSc‐ILD and SSc‐no ILD patients. (G) Heatmap of metabolomic data showing a significant upregulation of triglycerides (module 3) in cluster 3, which is comprised mainly of SSc‐ILD patients. (H) Volcano plot showing a significant increase in triglyceride levels in 20 SSc‐ILD patients vs 10 SSc‐no ILD patients. (I, J) Boxplots of CRP levels in SSc‐ILD compared to SSc‐no ILD patients in cohorts 1 and 2 respectively (*P*‐value ≤0.05). Results in C, D, F, I & J are depicted as boxes and whiskers plots defined by minimal, maximal, and median values in each dataset. The upper whisker extends from the hinge to the largest value no further than 1.5 * IQR from the hinge (where IQR is the interquartile range or distance between the first and third quartiles). The lower whisker extends from the hinge to the smallest value at most 1.5 * IQR of the hinge. Data beyond the end of the whiskers are called outlying points and are plotted individually. Statistical analyses were performed using the Kruskal–Wallis test for D and limma test for C, F, I & J. CRP, C‐reactive protein; FC, fold‐change; IQR, interquartile range; ILD, interstitial lung disease; SSc, systemic sclerosis.

Differentially expressed proteins were found in the plasma of the 9 SSc‐ILD patients from Cohort 1 (Fig. 3A) and a pathway analysis was performed on these proteins using the Reactome knowledge base (https://reactome.org/). Results showed associations with dysregulated innate immunity, hemostasis, and ECM organization signaling pathways (Fig. 3B). Plasma proteins associated with inflammation (FGA and C9) and platelet activation (APOB) were elevated in SSc‐ILD samples compared to SSc‐no ILD (Fig. 3C). The clotting factor F12 and APOC3 were found in lower levels in SSc‐ILD plasma (Fig. 3C). A significant increase in circulating levels of Gal‐3 (a lectin involved in fibrogenesis through initiation and amplification of the acute inflammatory response by recruiting macrophages to injury sites) was also observed in SSc‐ILD patients (Fig. 3D).

Significant differences were also observed in triglyceride and phosphatidylcholine levels between SSc‐ILD and SSc‐no ILD plasma (Cohort 1) (Fig. 3E). An increase in hexosylceramide (a sphingolipid integral to cell membranes) was observed in SSc‐ILD plasma (Fig. 3F). Reduced levels of lyso‐phosphatidylcholine (an endothelial cell activator) (Fig. 3E), arachidonic acid (omega‐6 polyunsaturated fatty acid associated with inflammatory and vascular processes), docosahexaenoic acid, and eicosapentaenoic acid (anti‐inflammatory omega‐3 polyunsaturated fatty acids) were also observed in patients with ILD compared to those with no ILD (Fig. 3F).

Interestingly, in the plasma of 20 other SSc‐ILD patients (Cohort 2), triglyceride levels were found to be significantly increased compared to those of 10 SSc‐no ILD patients (Fig. 3G,H).

However, no significant difference was found in relation to CRP levels, whether longitudinally (Fig. 3I) or linked to ILD (Fig. 3J).

### Absence of lung fibrosis associates with increased inflammatory peripheral blood cell phenotype

**Fig. 4:**
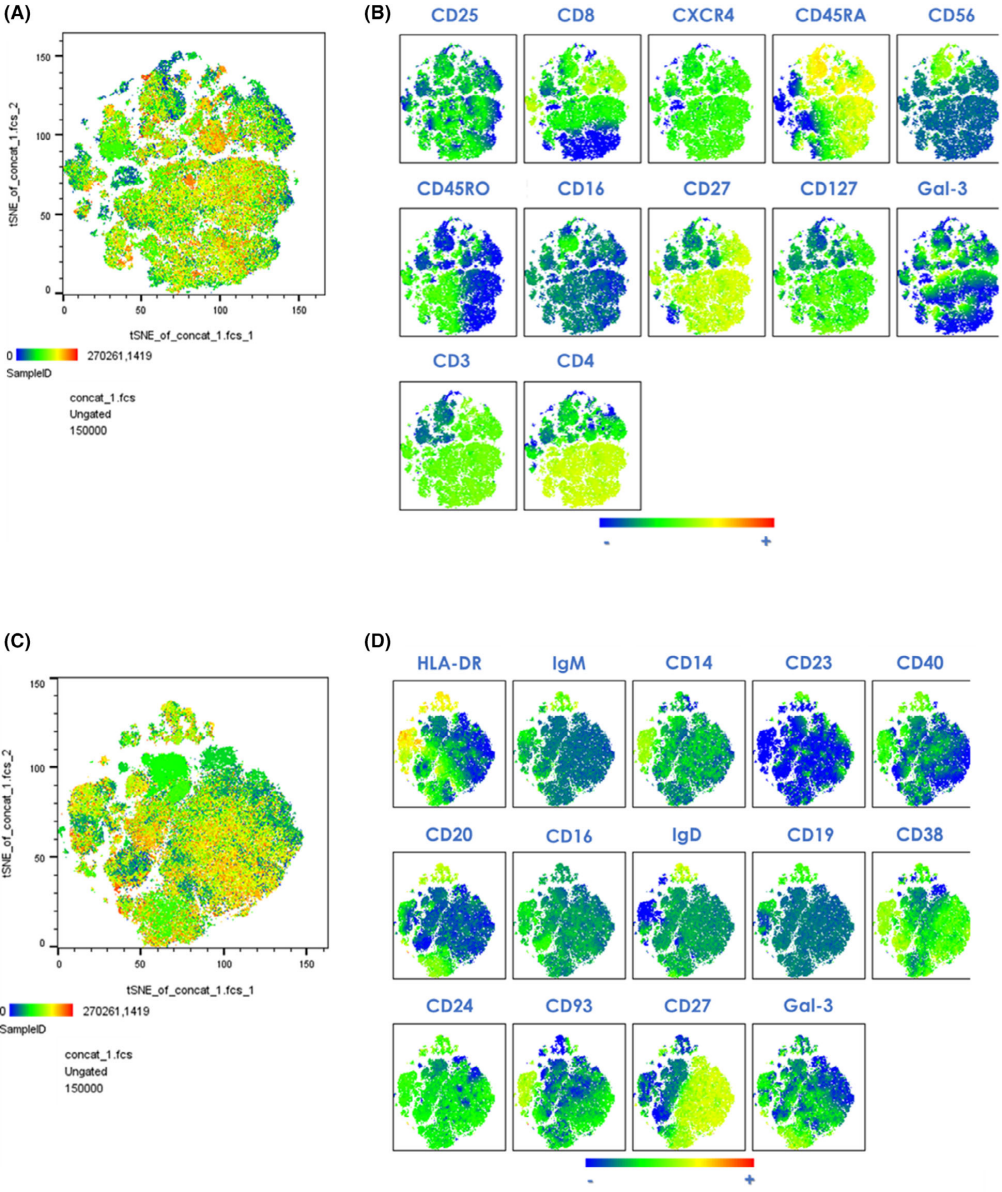
Immunophenotyping of peripheral blood cells (PBMCs) from SSc patients (*n* = 10) compared to healthy volunteers (HV; *n* = 5) – unsupervised analysis. (A, C) High‐dimensional data analysis on viable PBMCs from concatenated samples using the t‐Stochastic Neighbor Embedding plot (tSNE) plugin in FlowJo. (B, D) Individual graphs display density plots illustrating the global expression of each marker in the T/NK panel and B/mono panels (respectively). Yellow‐orange colors depict areas of high marker expression, whereas dark green‐blue areas indicate areas of lower marker expression. HV, healthy volunteer; ILD, interstitial lung disease; IQR, interquartile range; PBMC, peripheral blood mononuclear cells; SSc, systemic sclerosis.

**Fig. 5:**
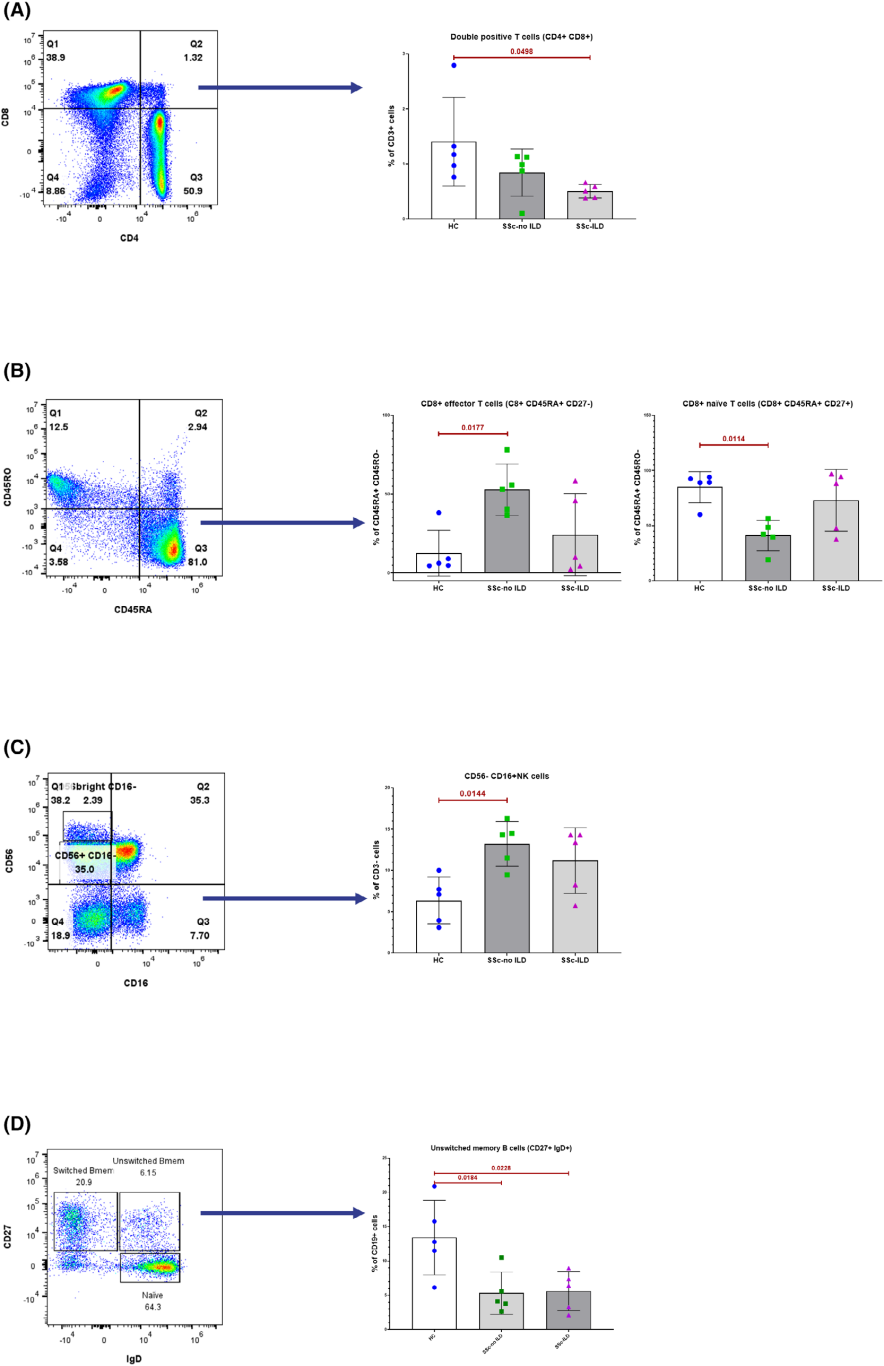
Immunophenotyping of peripheral blood cells (PBMCs) from SSc patients (*n* = 10) compared to healthy volunteers (HV; *n* = 5) – supervised analysis. (A–D) Density plots of the populations of interest are shown on the left and corresponding resulting boxplots from statistical analysis are shown on the right. (A) Double positive CD4 CD8 T cells are significantly decreased in SSc‐ILD patients compared to HVs (*P*‐value = 0.0498). (B) Increased frequency of CD8 effector T cells (*P*‐value = 0.0177) and decreased frequency of CD8 naïve cells (*P*‐value = 0.0114) are observed in SSc patients with no ILD compared to HVs. (C) CD56‐CD16+ NK cells are significantly increased (*P*‐value = 0.0144) in SSc patients with no ILD compared to HVs. A similar trend can be observed in SSc‐ILD patients. (D) Unswitched memory B cell frequency is significantly reduced in both SSc patient groups compared to HVs (*P*‐value = 0.0184 for SSc‐noILD patients and *P*‐value = 0.0228 for SSc‐ILD patients). Results in A–D are depicted on the right hand as boxplots and error bars represent standard deviation. Statistical analyses were performed using the One‐Way ANOVA test. HV, healthy volunteer; ILD, interstitial lung disease; IQR, interquartile range; NK, natural killer; PBMC, peripheral blood mononuclear cells; SSc, systemic sclerosis.

To detect potential immune cell abnormalities associated with SSc and the presence or absence of ILD, PBMCs from SSc patients were compared to those from HVs (Cohort 3) using flow cytometry. The clustering analysis of the immunophenotyping data using the tSNE and FlowSOM algorithms allowed the visualization of the global distribution of each cell surface marker (Fig. 4A–D). We subsequently performed a conventional manual gating strategy used to define immune cell populations (Fig. S1). Our results showed a reduced frequency of double positive CD4/CD8 T cells in SSc‐ILD patients compared to HVs (Fig. 5A). SSc‐no ILD patients, compared to HVs, presented with a lower frequency of CD8+ T naïve cells (CD8+ CD45RA+ CD27+) and an increased frequency of CD8+ effector T cells (CD45RA+ CD27‐) (Fig. 5B) and of CD56‐ CD16+ NK cells (Fig. 5C).

Regarding B cell subsets, we found decreased frequency of unswitched memory B cells only (IgD+ CD27+), in both SSc patient groups compared to HVs (Fig. 5D). No significant difference was observed in the myeloid compartment.

### Progression of established lung fibrosis associates with altered CX3CL1, CCL2, and triglycerides

**Fig. 6:**
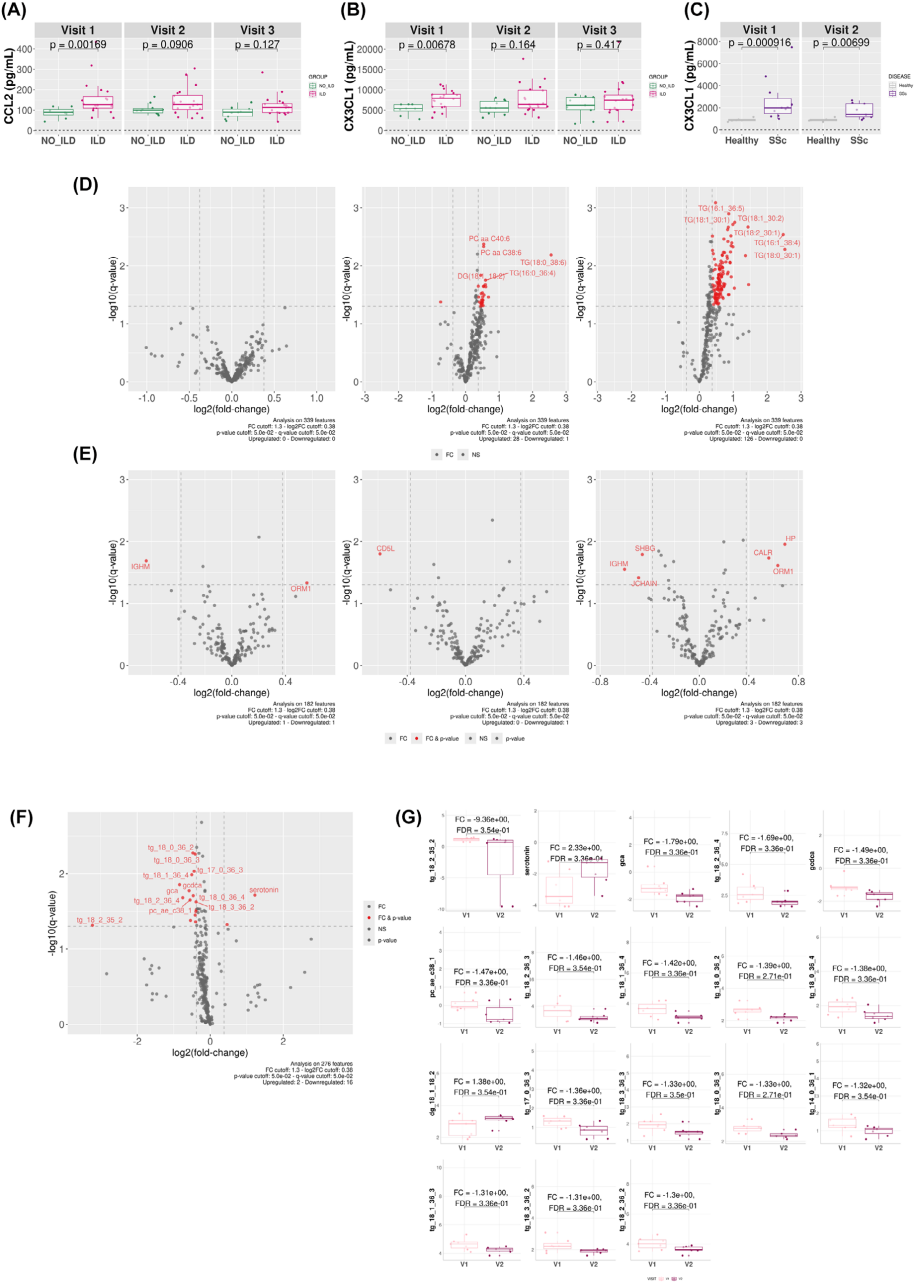
Longitudinal profiling of plasma cytokines, metabolites, and proteins. (A, B) Boxplots of CCL2 and CX3CL1 (respectively) levels in the plasma of SSc‐ILD vs SSc‐no ILD patients at each of their 3 visits for sample collection (cohort 2). (C) Circulating levels of CX3CL1 in the plasma of pooled SSc patients from cohort 1 compared to HVs at both their visits. (D) Volcano plots representing differentially expressed metabolites in the plasma of SSc‐ILD patients vs SSc‐no ILD patients at each of their 3 visits for sample collection (cohort 2). (E) Volcano plots representing differentially expressed proteins in the plasma of SSc‐ILD patients vs SSc‐no ILD patients at each of their 3 visits for sample collection (cohort 1). (F) Volcano plot representing differentially expressed metabolites after 6 months of follow‐up in the plasma of 9 SSc‐ILD patients (cohort 1). (G) Boxplot of the top 18 differentially expressed metabolites in SSc‐ILD patients compared to SSc‐no ILD patients (1.3 ≤ FC ≤ −1.3) and *P*‐value ≤0.05. Results in A–C & G are depicted as boxes and whiskers plots defined by minimal, maximal, and median values in each dataset. The upper whisker extends from the hinge to the largest value no further than 1.5 * IQR from the hinge (where IQR is the interquartile range or distance between the first and third quartiles). The lower whisker extends from the hinge to the smallest value at most 1.5 * IQR of the hinge. Data beyond the end of the whiskers are called outlying points and are plotted individually. Statistical analyses were performed using the Kruskal–Wallis test for A–C and limma test for G. CCL2, C‐C motif ligand 2; CX3CL1, fractalkine; ILD, interstitial lung disease; SSc, systemic sclerosis.

Following the comparison of SSc‐ILD patients to those with no ILD, we attempted to identify markers of ILD progression over the course of the disease. Starting from the analysis of plasma samples from the baseline collection timepoint of 30 SSc patients (Cohort 2), results showed increased levels of the chemokines CCL2 and CX3CL1 in SSc‐ILD patients as compared to SSc‐no ILD patients (Fig. 6A,B). This difference was not observed throughout follow‐up. CX3CL1 levels were also found to be increased in the plasma of the 12 SSc patients compared to the 5 HVs (Cohort 1) at both collection timepoints (Fig. 6C).

After an average 5‐year follow‐up of the 30 SSc patients (Cohort 2), triglycerides were found in significantly higher levels in SSc‐ILD plasma compared to SSc‐no ILD, as well as the levels of β‐alanine, a rate‐limiting precursor of carnosine, a major component of muscle (Fig. 6D).

Results also showed decreased levels of the proteins IGHM (immunoglobulin μ chain C region, which defines the IgM isotype) and JCHAIN (which favors the multimerization and secretion of IgM and IgA) in the 20 SSc patients with ILD progression (Cohort 2) (Fig. 6E). In the same patients, results showed increased levels of ORM1 (a key acute‐phase plasma protein) and HP (inhibits the deleterious oxidative activity of free hemoglobin) (Fig. 6E).

**Fig. 7:**
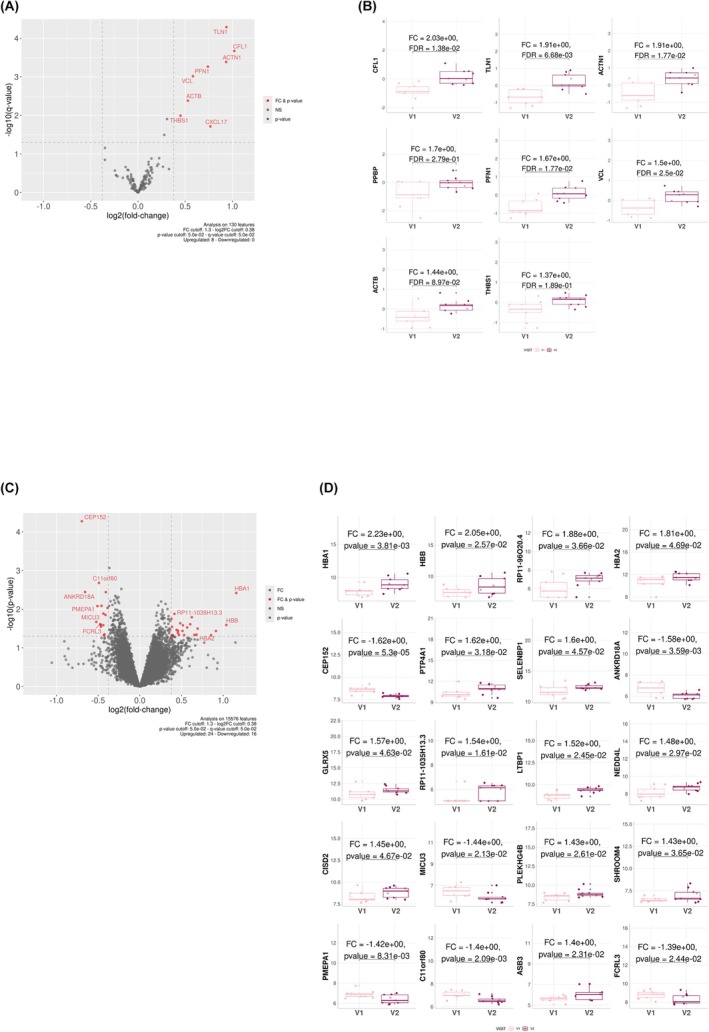
Longitudinal profiling of proteins and blood gene expression. (A) Volcano plot representing differentially expressed proteins after 6 months of follow‐up in the plasma of 9 SSc‐ILD patients (cohort 1). (B) Boxplots of the top 8 differentially expressed proteins in SSc‐ILD patients compared to SSc‐no ILD patients (1.3 ≤ FC ≤ −1.3 and *P*‐value ≤0.05). (C) Volcano plot representing differentially expressed genes after 6 months of follow‐up in the plasma of 9 SSc‐ILD patients (cohort 1). (D) Boxplots of the top 20 differentially expressed genes in SSc‐ILD patients compared to SSc‐no ILD patients (1.3 ≤ FC ≤ −1.3 and *P*‐value ≤0.05). Results in B and D are depicted as boxes and whiskers plots defined by minimal, maximal, and median values in each dataset. The upper whisker extends from the hinge to the largest value no further than 1.5 * IQR from the hinge (where IQR is the interquartile range or distance between the first and third quartiles). The lower whisker extends from the hinge to the smallest value at most 1.5 * IQR of the hinge. Data beyond the end of the whiskers are called outlying points and are plotted individually. Statistical analyses were performed using the limma test for B & D. ILD, interstitial lung disease; SSc, systemic sclerosis.

In addition, when focusing on the evolution of 9 SSc‐ILD patients (Cohort 1) over a shorter 6‐month follow‐up, the results showed a significant increase in serotonin (vasoactive mediator of inflammation) levels. A decrease in the levels of two biliary acids, gcdca and gca, and of triglycerides was also observed (Fig. 6F,G). Several proteins were found in significantly higher levels in SSc‐ILD after 6 months, notably cytoskeleton proteins (TLN1, CFL1, ACTN1, PFN1, VCL, ACTB), THBS1 (platelet aggregation, cell‐to‐matrix interactions) and CXCL17 (released by platelets, synthesis of ECM) (Fig. 7A,B). Interestingly, there was also a significant dysregulation in gene expression, with most notably a significant upregulation of LTBP1, which controls TGF‐β1 activation (Fig. 7C,D).

No significant correlation was found between the measured parameters and autoantibody profiles, comorbidities, overlap syndrome, gender, age, or treatment, which could further explain the progression of ILD in some patients but not in others.

## Discussion

We have conducted a comprehensive comparative analysis of blood and skin samples between 34 SSc‐ILD patients and 18 SSc patients without ILD using multi‐omics methods. This approach is particularly relevant in a multicompartmental disease such as SSc, notably to understand the determinants and potential markers of ILD, currently the most important cause of SSc‐related mortality. The findings build on other work suggesting an early phase of SSc‐ILD linked to immune dysregulation and a later stage that might reflect imprinted changes in fibrotic processes, leading to failed resolution and resulting intrinsic fibrosis leading to lung stiffness and ECM deposition.

The validity of our approach is supported by our demonstration of a significant upregulation of the type 1 interferon (T1 IFN) signature in SSc‐ILD patients. This finding corroborates previous observations [12, 13, 14, 15] that reported elevated levels of circulating IFNα in SSc patients compared to HVs, which correlated with the presence of ILD [14]. The detrimental role of IFNα in SSc pathogenesis was confirmed in a clinical trial in which patients received subcutaneous injections of IFNα or placebo. The trial was prematurely terminated because SSc patients showed significantly greater ILD worsening, measured by FVC% [15]. While it is established that the upregulated T1 IFN signature participates in SSc pathogenesis [13], our results suggest an important role in driving ILD progression, which could be impeded by blocking its receptor. The ongoing clinical trial of Anifrolumab targeting the IFNAR receptor with the aim of slowing down disease progression could confirm this hypothesis (NCT05925803) [16]. The upregulation of the IL‐6/Jak/STAT3 signaling pathway in the blood and skin of SSc‐ILD patients corroborates the fibrogenic role of IL‐6. This is of high interest since Tocilizumab, a monoclonal antibody targeting the IL‐6 receptor, has recently been approved for treatment of SSc‐ILD [17].

Our findings show that the chemokine CCL2 (secreted by monocytes, macrophages, fibroblasts, and endothelial cells) circulates at higher levels in the plasma of SSc‐ILD patients, thus supporting the importance of inflammation contributing to ILD development. Previous studies have established a correlation between the elevated levels of CCL2 and the increased severity and progression of lung fibrosis in SSc patients [18, 19, 20, 21], notably through its association with a high HRCT fibrosis score and poorer survival compared to healthy volunteers [21, 22]. Not only is CCL2 a chemoattractant for immune cells, it also stimulates TGF‐ß production in lung fibroblasts, which contributes to the accumulation of collagen and ECM [20, 23, 24]. Moreover, in response to CCL2, pro‐fibrotic fibroblasts have an increased expression of TGF‐ß receptors [21]. Our results, added to the data available in the scientific literature, lead to considering CCL2 as a potential candidate marker for the prediction of ILD progression. Levels of the CX3CL1 chemokine (secreted and expressed by epithelial and endothelial cells) were higher in the plasma of SSc‐ILD patients. In SSc patients, CX3CL1 is found in higher levels in endothelial cells of fibrotic skin and lung [25], and localized in reactive type II pneumocytes and airway epithelial cells [26]. Increased levels of soluble CX3CL1 have also been observed in both lung tissue and serum, which correlates with ILD progression and poor DLco [25, 26]. Our results corroborate this involvement of CX3CL1 in SSc‐ILD progression, likely through a role in the recruitment of immune cells.

Aligning with current interest in lipid mediators, which may play a role in lung fibrosis, our results showed altered levels of phosphatidylcholines and lyso‐phosphatidylcholines. The latter serve as precursors of lysophosphatidic acid (LPA), which is found in increased levels in the bronchoalveolar lavage of IPF patients [27]. LPA triggers stress fiber formation in normal human bronchial epithelial cells, leading to transforming growth factor beta (TGF‐β) activation [28]. LPA signaling through its receptor LPA1 mediates fibroblast recruitment and vascular leakage [29]. Our findings support the current interest in LPA with the LPA1 antagonist BMS‐986278 in a phase 3 clinical trial in IPF and ILD (NCT06003426 and NCT06025578 respectively).

Proteins implicated in the innate immune response, namely APOB, C9, Gal‐3, FGA, ORM, and HP, are increased in SSc‐ILD plasma, supporting the possibility that they may predict ILD progression. APOB is involved in platelet activation [30]; C9 is a component of the membrane attack system of the complement system, which has already been implicated in SSc pathogenesis [31]; Gal‐3 is involved in fibrogenesis via macrophage recruitment, TGF‐β production, and fibroblast proliferation [32]; FGA, ORM, and HP are acute‐phase proteins, whose concentrations increase in response to inflammation and participate in the initiation of tissue remodeling following injury [33, 34, 35, 36]. The association of increased levels of the acute‐phase protein, C‐reactive protein, with an elevated risk of mortality and of the likelihood of developing ILD in SSc has previously been described [37].

Alterations of the adaptive immune system are consistent with the immune dysregulations underlying SSc and its complications. In SSc‐ILD compared to healthy volunteers, we found a significantly reduced frequency of CD4/CD8 double positive (DP) T cells. DP T cells are believed to be chronically activated CD8 T cells with the effector functions of the CD4 lineage, rendering them highly inflammatory [38]. In SSc‐no ILD patients compared to HVs, we found an increased frequency of CD56‐ CD16+ NK cells and of CD8 T effector cells, while CD8 naïve T cell frequency was reduced. This subtype of NK cells is the most cytotoxic [39]. In a study by Fuschiotti and colleagues (2009) [40], effector CD8+ T cells were found to be more frequent in SSc patients, although other studies have shown either no difference or decreased levels of both T cell subtypes in SSc patients as compared to HVs [39]. Our results indicate lower cellular inflammatory activity in tissues of the periphery of SSc‐ILD patients, which may be a result of ongoing immunosuppressive therapies; SSc patients with no ILD showed a more inflammatory cellular phenotype. We further show a decrease in unswitched memory B cells (IgD+ CD27+) in both SSc patient groups, concordant with available literature, indicating that disturbance in immunoregulation may be partly due to the imbalance of tolerogenic (unswitched) and activated (switched) memory B cells [41].

When focusing on ILD progression, our observation of elevated triglyceride levels in the plasma of SSc‐ILD patients over an average 5‐year follow‐up has not been previously reported and represents a potentially exciting finding. In parallel, reduced levels of APOC3, a protein that delays the catabolism of triglyceride‐rich particles, are consistent with the triglyceride increase. Higher levels of triglycerides have previously been observed in SSc patients compared to HVs [30, 42]. Triglycerides are known indicators of chronic inflammation, notably of cardiovascular risk, associated with age or inadequate diet [43, 44, 45]. They are abundant circulating lipids, stored in droplets within the endoplasmic reticulum (ER). They are involved in the induction of ER stress [46], which can induce apoptosis of epithelial cells and their transition to myofibroblasts [47]. Their action can also induce macrophage polarization into an M2 phenotype, secreting profibrotic mediators [47]. Higher levels of triglycerides are observed in SLE, another autoimmune systemic disease, highlighting that they may be indicative of inflammatory‐mediated pathological mechanisms [48]. Triglyceride levels are included in diagnostic criteria for hemophagocytic lymphohistiocytosis (HLH), a life‐threatening hyper‐inflammatory disorder, as a marker of poor outcome [49], opening the way for considering triglycerides and other lipids as potential predictive markers of disease progression.

Furthermore, over 6 months of follow‐up, we found higher levels of serotonin in SSc‐ILD plasma, which has been suggested to play a role in driving fibrosis by promoting platelet aggregation and vasoconstriction [50, 51]. Our results also show elevated levels of CXCL17, a protein that contributes to ECM contraction through regulating human fibroblast growth by activation of PDGF. It has previously been found in higher levels in SSc patients compared to HVs [52, 53]. The observed increase in several cytoskeleton proteins indicates an increase in profibrotic processes through their role in endothelial to mesenchymal transition [54, 55] and in the enhanced interplay between components of connective tissues, such as fibroblasts, surrounding matrix, vascular, and immune cell populations [56]. Elevated levels of THBS1 (thrombospondin), a protein released following platelet activation and a substrate for coagulation Factor XIII [57], were observed. THBS1 is also known to be an endogenous activator of latent TGFβ during matrix contraction to enhance the contractile activity of pathological SSc fibroblasts [58]. It could be considered a marker of ongoing tissue injury driving ILD progression. At the transcriptomic level, we found an increase in the expression of LTBP1, whose transcribed protein binds latent TGF‐β and enables its deposition to be localized within the ECM in proximity to TGF‐β expressing cells (e.g., epithelial and endothelial cells) [59]. This highlights potential mechanistic links with local TGF‐β activation at the epithelial‐endothelial barrier in the lung that could explain how these manifestations lead to fibrosis.

The major strengths of this study include the use of well‐defined patient cohorts with careful and clinically relevant stratification; the unbiased multi‐omic analysis using platform technologies; the documented disease progression over short (6 months) or long (5 years) time frames in the longitudinal studies; and the inclusion of patients with established SSc in the cross‐sectional cohort. This inclusion allowed for a robust comparison between SSc‐ILD patients and those without ILD, as the latter were outside the time frame for ILD development.

The main limitation of our study is its sample size, which was inevitably small due to strict patient stratification. Other limitations include the distinct technical processing required for sample analyses that could impact direct comparison and integration of results; the different normalization methods used across platforms; and the potential confounding influences related to differences in disease duration, intrinsic disease diversity, and background treatment, such as immunosuppressants, which may influence the results.

In conclusion, our results build on the existing literature, confirming the involvement of pro‐inflammatory mechanisms in SSc‐related ILD (Type 1 Interferon, CX3CL1, CCL2), as well as pro‐fibrotic (CXCL17, THBS, LTBP1). At the cellular level, our results indicate lower inflammatory activity in SSc‐ILD patients, which may be a result of ongoing immunosuppressive therapies. A novel finding of our study is the correlation between elevated triglyceride levels and ILD progression, which may be linked to fibrogenesis via the role of triglycerides in endoplasmic reticular stress. While blood and skin samples may not be the most representative for studying lung fibrosis, they remain the most accessible in clinical practice for identifying markers of disease progression.

Our study is the first to demonstrate that discrete biological differences can be identified using a multi‐omic approach in small patient cohorts. The feasibility and potential of this approach suggest that future comparative multi‐omic profiling studies will further elucidate the pathobiology of SSc‐ILD. The identification of candidate markers associated with ILD progression offers valuable insights into the underlying pathophysiological mechanisms, potentially supporting the design of novel treatments for severe forms of SSc.

## Materials and methods

### Study design and patient selection

Fifty‐two SSc patients and five healthy volunteers (HVs, aged between 45 and 60 years) signed an informed consent for the use of their clinical data and biological samples for research purposes. This study complies with the Declaration of Helsinki. It received ethics approval from the NHS Research and Ethics Committee (REC); London‐Hampstead (IRAS 270295) and London‐Fulham (IRAS 279682). Sample collection was carried out at the Royal Free Hospital, London UK, between January 2000 and February 2024.

**Fig. 8:**
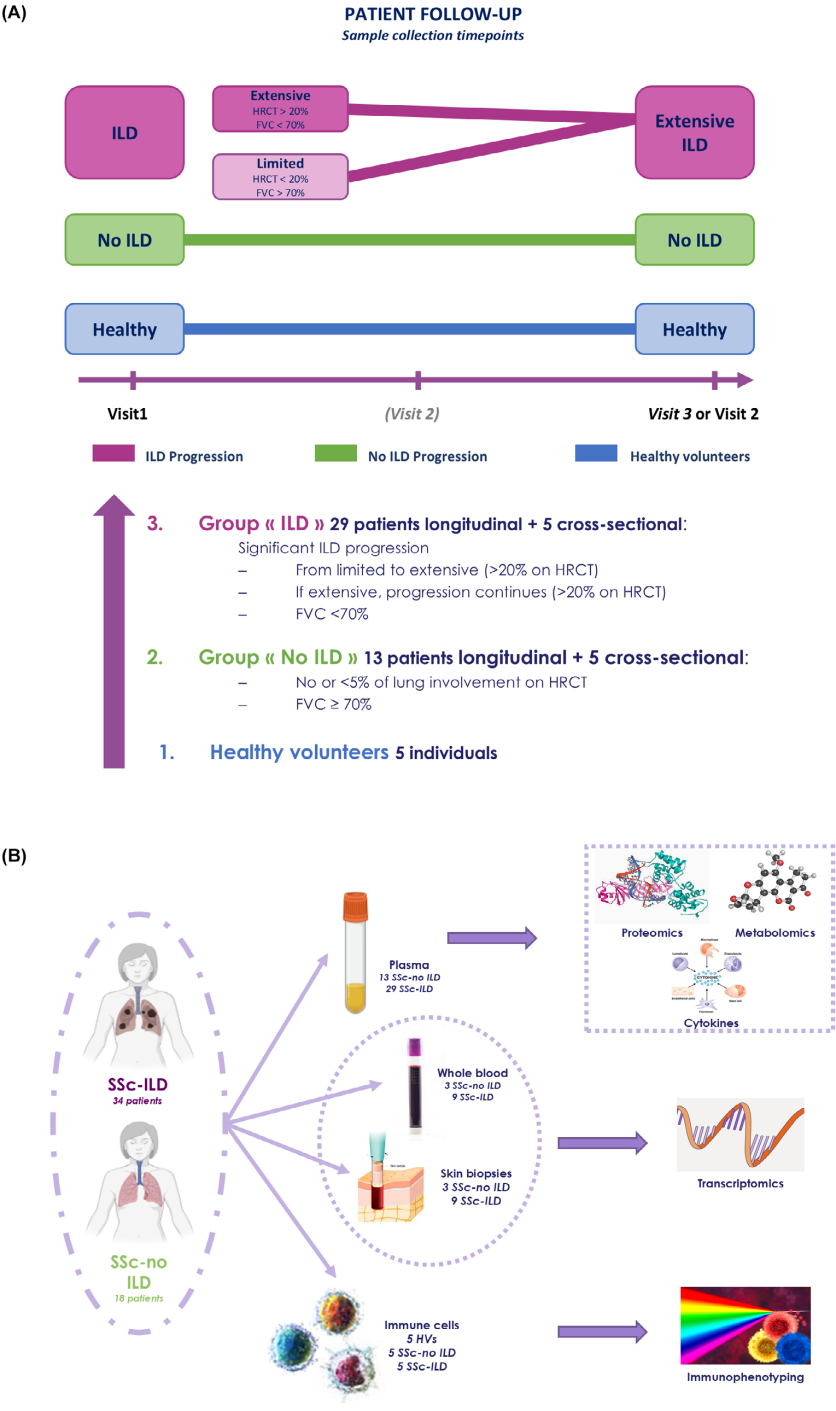
Study design and sample collection. (A) Schematic showing the study design, where participants were divided into 3 sub‐groups depending on the presence of ILD and its progression over time. (B) Image illustrating the different sample types collected from study participants and the analyses performed on those samples. ILD, interstitial lung disease.

To be included, SSc patients had to fulfill the ACR/EULAR Classification Criteria for Systemic Sclerosis and had to be positive for ACA, ARA and/or anti‐Scl‐70 autoantibodies. Patients were divided into two groups based on ILD progression over follow‐up: “SSc‐no ILD” patients had low or no clinical signs of ILD over time, characterized by less than 5% lung involvement on high resolution computed tomography (HRCT), and “SSc‐ILD” patients presented with visible ILD progression, characterized by 20% or more lung involvement (Fig. 8A).

Recorded clinical data include demographic parameters, pulmonary function tests (FVC and diffusing capacity of the lungs for carbon monoxide, DLCO), HRCT scans, comorbidities, overlapping diseases, and treatments (Table 2).

**Table 2:** Summary of analyses performed for each cohort and the samples concerned. HV, healthy volunteers; ILD, interstitial lung disease; SSc, systemic sclerosis.

	Cohort 1 (prospective)	Cohort 2 (retrospective)	Cohort 3 (cross‐sectional)	Total
Follow‐up	6 months	~5 years	–	
ILD	9	20	5	34
No ILD	3	10	5	18
HV	5	‐	5	5
Total	17	30	15	57
Samples	29	90	15	
Analysis	Transcriptomics Proteomics Metabolomics Cytokines	Proteomics Metabolomics Cytokines	Immunophenotyping	

Plasma samples, whole blood, skin biopsies, and peripheral blood mononuclear cells were available for multi‐omic analysis (Fig. 8B).

### Sample analyses

#### Analysis of soluble mediators in plasma samples

##### Metabolomics

Metabolomics was performed on plasma samples using tandem mass spectrometry with the Biocrates MxP® Quant 500 Kit (Biocrates, Innsbruck, Austria), which enables the targeted analysis of up to 630 metabolites from 26 biochemical classes. Data acquisition was performed using an Acquity UPLC I‐Class system (Waters, Guyancourt, France) coupled to a 6500 QTRAP^®^ instrument (AB Sciex, Darmstadt, Germany). Data preprocessing steps were performed with the Biocrates MetIDQ software and R internal scripts.

##### Proteomics

Plasma samples underwent depletion to remove the most abundant proteins (Multiple Affinity Removal System™ columns, 4.6 × 50 mm; Agilent, Palo Alto, CA, USA). The recovered proteins were protected and digested (iST‐NHS kit, PreOMICS, Planegg, Germany); peptides were differentially labeled using Tandem Mass Tags (TMT11plex isobaric label kit, ThermoFischer, Montigny‐le‐Bretonneux, France), mixed, and cleaned up (iST‐NHS kit, PreOMICS). The Liquid Chromatography‐Electrospray Ionization‐Mass Spectrometry (LC‐ESI‐MS/MS) analysis was performed using an EASY‐nLC 1200 system coupled to a quadrupole Orbitrap (Q Exactive HF, ThermoFischer) mass spectrometer, equipped with a Nano‐EASYspray ion source and driven by Xcalibur 4.1 instrument control (ThermoFischer).

Proteome Discoverer software version 2.5 (PD 2.5) was used to analyze and quantify TMT‐based data, sequest scoring algorithm allowing identification of proteins based on database searching using the human SwissProt DB (release 2022_10).

##### Galectin‐3 measurements

Concentrations of Galectin‐3 (Gal‐3) were measured using the Simple Plex Human Galectin‐3 Cartridge (ref. SPCKB‐PS‐000490, Bio‐techne, Noyal Châtillon sur Seiche, France). The plasma samples were assessed using the automated ELLA instrument (BioTechne). Data were preprocessed using the Simple Plex Explorer Software 4.1.0.22 (BioTechne).

##### Cytokine measurements

Meso Scale Discovery (MSD, Puteaux, France) custom U‐plex plates, R‐PLEX, and S‐Plex human assays were used to measure the concentrations of 27 cytokines/chemokines (*CCL2*, *CCL3*, *CCL4*, *CCL5*, *CCL17*, *CCL18*, *CXCL8*, *CXCL10*, *CX3CL1*, *GM‐CSF*, *IFNα*, *IFN‐γ*, *IL‐1β*, *IL‐2*, *IL‐4*, *IL‐5*, *IL‐6*, *IL‐10*, *IL‐12p70*, *IL‐13*, *IL‐17A*, *PDGF‐A*, *TGF‐β*, *TNF‐α*, *TSLP*, *VEGF‐α*, and *YKL‐40*) in the plasma of study participants, in accordance with the manufacturers' recommendations. MSD plates were read on a Mesoscale SQ120 (MSD) instrument. Data were analyzed using the MSD Workbench v4.0 software.

#### Immunophenotyping

Peripheral mononuclear cells were stained with fluorochrome‐coupled antibodies (Table S2).

Cells were analyzed using a CytoFLEX LX Flow Cytometer (Beckman Coulter, Villepinte, France, product N°C40324) acquiring at least 250 000 events/sample. Data analysis was performed using the FlowJo v.10.10.0 software (Becton, Dickinson & Company), using both supervised and unsupervised (tSNE and FlowSom algorithms) analyses.

For multidimensional analysis, the data were pregated to remove dead cells and debris, selection of cells of interest using the FlowJo 10.10.0 software, as shown in (Fig. S1A). Manual gating was performed in FlowJo, as shown in Fig. S1B–F. Unsupervised clustering was performed using the tSNE and FlowSOM algorithms (Fig. 3A–D). Frequencies of different populations were exported from FlowJo and analyzed using Prism 9 (GraphPad Software, San Diego, CA, USA).

#### Transcriptomics—RNAseq

Global gene expression profiling was performed in whole blood and skin biopsies. Punch biopsies (4 mm) were taken from subjects under local anesthetic from the volar aspect of the left or right mid‐forearm and, where possible, from clinically involved skin. Repeat biopsies were taken from the same anatomical site. The biopsy was then immediately transferred to prelabeled and prefilled containers containing RNAlater for transcriptomics. The biopsy sample was then incubated overnight (a minimum of 16 h and maximum of 24 h) at 4 °C, and then stored at −80 °C until analysis. Then, total RNA was extracted using the miRNAeasy Mini Kit (Qiagen) according to the manufacturer's protocol.

The samples were stored in RNAlater prior to RNA extraction and were sequenced on the NovaSeq instrument (Illumina, San Diego, USA). Resulting data were converted to fastq files using Illumina's bcl2fastq conversion software v2.19.

#### Data analysis

##### Differential expression analysis

Differential expression analysis of gene, protein, and metabolite levels was performed using a linear regression model (lmfit function from the limma R package) on the vst transformation gene expression dataset. Resulting *P*‐values were adjusted for multiple hypothesis testing and filtered to retain differentially expressed genes with a False Discovery Rate (FDR) adjusted <0.1 and a Fold‐Change (FC) ≥ 1.3.

Cytokine levels were analyzed using a Wilcoxon test.

##### Enrichment analysis

Enrichment analysis was performed by applying a two‐tailed Fisher‐exact test against different sources of gene modules or pathways: the fGSEA approach based on Hallmark gene sets, and the canonical pathways from Ingenuity Pathway Analysis (IPA).

Rinchai *et al*. (2021) have developed an R package, BloodGen3Module, which allows comparisons between study groups at a module level. They established a repertoire of 382 gene modules covering 14 168 transcripts based on the co‐clustering observed across 16 different pathological states (autoimmune and infectious diseases, primary immune deficiency, cancer and pregnancy) [60, 61]. From these 382 modules, a reduced level with 38 variables was built (A1 to A38) constituted by module sets and functionally annotated pathway, ontology, and literature term enrichment.

Changes in blood and skin transcript abundance in SSc‐ILD patients compared to SSc‐no ILD patients with a fold change cutoff = 1.3 and an FDR‐adjusted *P*‐value <0.05 are represented on the fingerprint grid plot. An increase in transcript abundance for a given module is represented by a red spot and a decrease by a blue spot.

Among the 382 modules, six modules are annotated as “interferon.” For each of these, an interferon–protein interaction network is inferred, using the Pearson correlation test. Only significant and relevant correlation levels are considered (*P*‐value <0.05 and ρ ≥ 0.5, respectively).

## Author contributions

SB: planned experiments, performed experiments, analyzed data, wrote the paper. PS: analyzed data, contributed to writing the paper. AC, VO: substantial contributions to the conception and design of the work, and to the collection of patient samples. EN, PBSH, GL: planned experiments, critical review of the paper. LA, IW, ALG, MC: performed experiments. AA, MP: critical review of the work and final approval of the version to be published. JA, PM: substantial contributions to the conception and design of the work, critical review of the work, and final approval of the version to be published. DA, CPD: substantial contributions to the conception and design of the work, and to the collection of patient samples; critical review of the work and final approval of the version to be published.

## Conflict of interest

CPD has received consultancy fees from Abbvie, Janssen, GlaxoSmithKline, Boehringer Ingelheim, Roche, Novartis, Certa, Mitsubishi, Quell, and research grants to the Institution from Abbvie, GlaxoSmithKline, and Servier. SB, PS, EN, PBSH, GL, LA, IW, ALG, MC, AA, and JA are employees of Servier.

## Supporting information


**Fig. S1.** Gating strategy for analysis of PBMC panels.
**Table S1.** Summary of main results from the multi‐omic analyses performed.
**Table S2.** Descriptive summary of PBMC panels.

## Data Availability

The data that support the findings of this study are available from the corresponding author (selena.bouffette@servier.com) upon reasonable request.
