# Supplementary material for: Multi‐omic analysis identifies biological processes underlying progressive interstitial lung disease in systemic sclerosis
Source: FEBS J. 2025 Jul 7;292(22):6054–74. doi: 10.1111/febs.70177 (PMC12631162; doi:10.1111/febs.70177)
Supplement: Supplementary file 1 — Fig. S1. Gating strategy for analysis of PBMC panels. Table S1. Summary of main results from the multi‐omic analyses performed. Table S2. Descriptive summary of PBMC panels. [file FEBS-292-6054-s001.pdf]

Sample type	*Patient cohorts*	*Biological analyses*	*Main results*	*Relevance to disease*	*References*
Whole blood<br>Skin	Cohort 2<br>*9 SSc-ILD vs 3 SSc-noILD*	**Transcriptomics**	Upregulated IFN signature in SSc-ILD	Correlated with ILD presence in SSc patients; IFNα treatment worsened ILD.	Eloranta et al., 2010;<br>Black et al., 1999
			Upregulation of LTBP1	Its transcribed protein binds latent TGF-β and enables its deposition to be localized within the ECM in proximity to TGF-β expressing cells (e.g. epithelial cells).	Budi et al., 2021
Plasma	Cohorts 1 & 2<br>*20 SSc-ILD vs 10SSc-noILD*<br>*9 SSc-ILD vs 3 SSc-noILD*	**Proteomics**	Increased levels of C9, APOB, Gal-3	C9: component of complement system<br>APOB: platelet activation<br>Gal-3: macrophage recruitment, TGF-β production and fibroblast proliferation	Pellicano et al., 2022 ;<br>Ferraz-Amaro et al., 2021 ;<br>Bouffette et al., 2023
			Increased levels of FGA, ORM1 and HP	Acute phase proteins, increase in response to inflammation, initiation of tissue remodelling following injury	Luyendyk et al., 2019
			Increased levels of cytoskeleton proteins	Role in endothelial to mesenchymal transition and in the enhanced interplay between components of connective tissues, such as fibroblasts, surrounding matrix, vascular and immune cell populations.	Ciszewski 2021;<br>Wiktorska 2023;<br>Langevin et al., 2011
			Increased levels of THSB1	Released following platelet activation and a substrate for coagulation Factor XIII. Known to be an endogenous activator of latent TGFβ during matrix contraction to enhance contractile activity of pathological SSc fibroblasts.	Dardik et al., 2005 ;<br>Feng & Gerarduzzi, 2020
			Increased levels of CXCL17	Contributes to ECM contraction through regulating human fibroblast growth by activation of PDGF. It has been previously found in higher levels in SSc patients compared to HVs	Scambi et al., 2010;<br>Shimada et al., 2020
			Decreased levels of F12, APOC3	F12: Clotting factor<br>APOC3: protein that delays the catabolism of triglyceride-rich particles; involved in dysregulated metabolic pathway in IPF patients.	Bargagli et al., 2020
			Decreased levels of IGHM and JCHAIN	IGHM: immunoglobulin μ chain C region, which defines the IgM isotype;identified as potential candidates for novel diagnostic biomarkers of IPF.<br>JCHAIN: favours the multimerization and secretion of IgM and IgA; part of the gene signature of SSc skin plasma cells.	Yang et al. 2012;<br>Jia et al., 2023
		**Metabolomics**	Increased levels of triglycerides and phosphatidylcholines	Elevated levels of triglycerides in SSc patients compared to HVs. They are stored in droplets formed in the endoplasmic reticulum (ER) where they are involved in the induction of ER stress, which can induce apoptosis of epithelial cells and their transition to myofibroblasts. It can also induce macrophage polarisation into an M2 phenotype, secreting pro-fibrotic mediators.	Abrarovna, 2022;<br>Ferraz-Amaro et al., 2021;<br>Seeliger et al., 2022;<br>Kropski & Blackwell, 2018
			Increased levels of β-alanine	A rate-limiting precursor of carnosine, a major component of muscle; beta-alanine metabolism was upregulated in fibrotic lung tissues of bleomycin-treated mice.	Washimkar et al., 2023
			Increased serotonin levels	Role in driving fibrosis, through promoting platelet aggregation and vasoconstriction.	Sagonas 2022;<br>Petric et al., 2021
			Decreased levels of lyso-phosphatidylcholines	Precursor of LPA, which is found in increased levels in the BAL of IPF patients. Triggers stress fiber formation in normal human bronchial epithelial cells, leading to transforming growth factor beta (TGF-β) activation and its signalling through its receptor LPA1 mediates fibroblast recruitment and vascular leakage.	van der Aar et al., 2019 ;<br>Neighbors et al., 2023;<br>Tager et al., 2008
		**Cytokines**	Increased CCL2 levels	Correlated with ILD severity and progression; CCL2 stimulates TGF-ß production in lung fibroblasts.	Assassi et al., 2013;<br>King, Abraham & Stratton, 2018;<br>Buechler et al., 2021
			Increased CX3CL1 levels	Increased levels in both lung tissue and serum, and correlated with ILD progression, role in immune cell recruitment.	Hasegawa et al., 2005;<br>Hoffman-Vold et al., 2018
PBMCs	Cohort 3<br>*5 HVs vs 5 SSc-ILD vs 5 SSc-noILD*	**Immunophenotyping**	Reduced frequency of DP T cells in SSc-ILD vs HV	Chronically activated CD8 T with the effector functions of the CD4 lineage, rendering them highly inflammatory.	Hess et al., 2023
			Increased frequency of CD8 effector T cells and reduced frequency of CD8 naïve T cells in SSc-no ILD vs HV	Frequency of effector CD8+ T cells was increased in SSc patients, while other studies showed either no difference or decreased levels of both T cell subtypes in SSc patients compared to HVs.	Almeida et al., 2015
			Increased frequency of CD56- CD16+ NK cells in SSc-no ILD vs HV	Most cytotoxic NK subset.	Almeida et al., 2015
			Reduced frequency of unswitched memory B cells in both SSc groups vs HV	In SSc, disturbance in immunoregulation may be partly due to the imbalance of tolerogenic (unswitched) and activated (switched) memory B cells.	Simon et al., 2016

Supplementary table 1: Summary of main results from the multi-omic analyses performed.

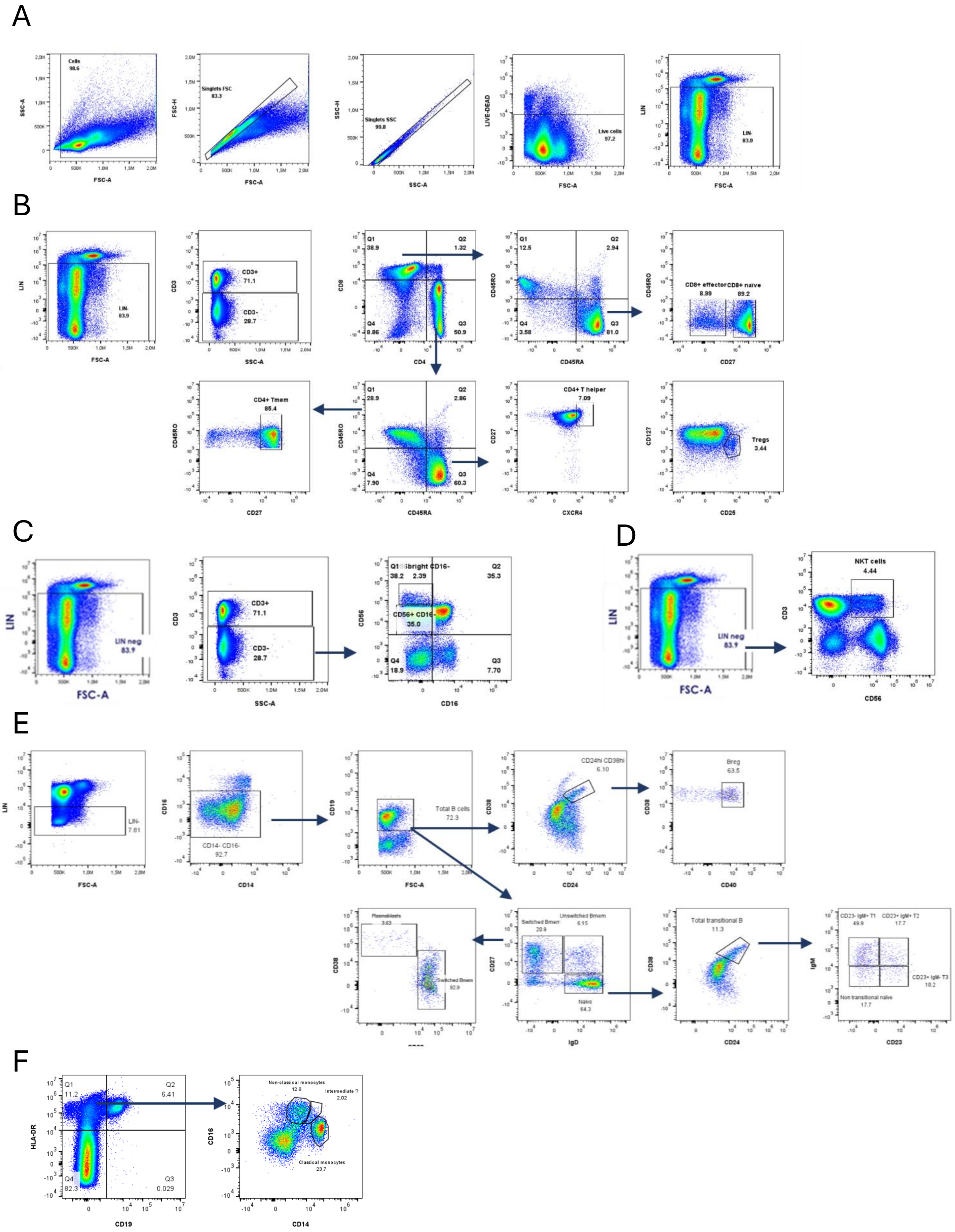

<u>Supplementary figure 1:</u> Gating strategy for analysis of PBMC panels.

(A) Manual gating strategy to remove debris and dead cells. Approach for manual gating for the major PBMC subsets: T cells (B), NK cells (C), NKT cells (D), B cells (E) and monocytes (F).

Panel	Marker	Fluorophore	Reference	Other name	Purpose
T/NK	CD45RA	BUV395	740298 (BD)		T cell differentiation, marker for naïve and effector T cells
	CD8	BUV496	741161 (BD)		CD8 T cell lineage
	CD127	BV421	351309 (BioLegend)	IL-7Rα	T cell differentiation
	**CD3**	BV510	300447 (BioLegend)		Pan T cells
	**CD4**	BV605	300555 (BioLegend)		CD4 T cell lineage
	**CD25**	BV785	302637 (BioLegend)	IL-2R α chain	Treg marker; activation marker expressed on T cells, B cells, monocytes/macrophages
	**CXCR4**	PerCP/Cy5.5	306515 (BioLegend)	CD184	T cell migration and activation
	**CD56**	APC	362503 (BioLegend)	NCAM *(Neural cell adhesion molecule)*	Pan NK cells
	**CD45RO**	AF700	304217 (BioLegend)		T cell differentiation, activated and memory T cell marker
T/NK & B/Mono	**Gal-3**	PE	126706 (BioLegend)	Mac-2	Lectin expressed on monocytes and leucocytes
	**CD27**	PE-Dazzle 594	562297 (BD)		T and B cell differentiation, expressed on T cells, B cells, NK cells
	**CD16**	BUV805	748850 (BD)		Monocyte and NK cell differentiation
	**Live/Dead**	Maleimide iFluor 860	1408 (AAT Bioquest)		
B/Mono	**CD23**	BUV395	743432 (BD)	FcεRII	Low-affinity receptor for IgE on B cells, transitional state
	**CD14**	BUV496	750381 (BD)		Monocyte differentiation
	**CD40**	BUV661	741624 (BD)		B cell activation
	**IgD**	BV421	348226 (BioLegend)		B cell differentiation, marker of immaturity
	**CD19**	BV510	302241 (BioLegend)		B cell lineage
	**CD38**	BV605	303531 (BioLegend)	Cyclic ADP ribose hydrolase	Monocyte, DC, T cells, B cells and NK cell activation/differentiation
	**IgM**	BV650	314525 (BioLegend)		B cell differentiation, marker of immaturity
	**HLA-DR**	BV785	307641 (BioLegend)		Activation marker expressed on B cells, T cells, APCs; marker for monocyte and DC lineage
	CD24	PerCP/Cy5.5	561647 (BD)		B cell differentiation
	CD93	APC	336119 (BioLegend)		B cell precursor marker
	CD20	AF700	302322 (BioLegend)		B cell lineage

Supplementary table 2: Descriptive summary of PBMC panels.
